# Classifying coherent versus nonsense speech perception from EEG using linguistic speech features

**DOI:** 10.1038/s41598-024-69568-0

**Published:** 2024-08-14

**Authors:** Corentin Puffay, Jonas Vanthornhout, Marlies Gillis, Pieter De Clercq, Bernd Accou, Hugo Van hamme, Tom Francart

**Affiliations:** 1 Department Neurosciences, KU Leuven, ExpORL, Leuven, Belgium; 2 Department of Electrical engineering (ESAT), KU Leuven, PSI, Leuven, Belgium

**Keywords:** EEG decoding, Deep learning, CNN, Linguistics, Auditory system, Biomedical engineering, Computational science

## Abstract

When a person listens to natural speech, the relation between features of the speech signal and the corresponding evoked electroencephalogram (EEG) is indicative of neural processing of the speech signal. Using linguistic representations of speech, we investigate the differences in neural processing between speech in a native and foreign language that is not understood. We conducted experiments using three stimuli: a comprehensible language, an incomprehensible language, and randomly shuffled words from a comprehensible language, while recording the EEG signal of native Dutch-speaking participants. We modeled the neural tracking of linguistic features of the speech signals using a deep-learning model in a match-mismatch task that relates EEG signals to speech, while accounting for lexical segmentation features reflecting acoustic processing. The deep learning model effectively classifies coherent versus nonsense languages. We also observed significant differences in tracking patterns between comprehensible and incomprehensible speech stimuli within the same language. It demonstrates the potential of deep learning frameworks in measuring speech understanding objectively.

## Introduction

Electroencephalography (EEG) is a non-invasive method that can be used to study brain responses to sounds. Traditionally, unnatural periodic stimuli (e.g., click trains, modulated tones, repeated phonemes) are presented to listeners, and the recorded EEG signal is averaged to obtain the resulting brain response and to enhance its stimulus-related component^3,31,33^. These stimuli do not reflect everyday human natural speech, as they are repetitive, not continuous, and are thus processed differently by the brain^24^. Although these measures provide valuable insights about the auditory system, they do not provide insights about speech intelligibility. To investigate how the brain processes realistic speech, it is common to model the transfer function between the presented speech and the resulting brain response^11,18^. Such models capture the time-locking of the brain response to certain features of speech, often referred to as neural tracking. Three main model types are being used to measure the neural tracking of speech: (1) a linear regression model that reconstructs speech from EEG (backward modeling); (2) a linear regression model that predicts EEG from speech (forward modeling); and (3) classification tasks that associate synchronized segments of EEG and speech among multiple candidate segments^13,15,35^. For forward and backward models, the correlation between the ground truth and predicted/reconstructed signal provides the measure of neural tracking, while for the classification task, classification accuracy is utilized. Estimations of neural tracking with such models can be used to measure speech intelligibility.^40^ showed a strong correlation between the neural tracking estimation obtained with linear models and speech intelligibility behavioural measurements.

To investigate how the brain processes speech, research has focused on different features of speech signals, which are known to be processed at different stages along the auditory pathway. Three main classes have hence been investigated:

- Acoustics (e.g., spectrogram, speech envelope^18^, f0^34,39^)
- Lexical segmentation features (e.g., phone onsets, word onsets,^17,30^)
- Linguistics (e.g., phoneme surprisal, word frequency,^7,8,20,28,36,42^)

As opposed to neural tracking studies using broad features that carry mostly acoustic information, we here select linguistic features to narrow down our focus to speech understanding. Linguistic features of speech reflect information carried by a word or a phoneme, and their resulting brain response can be interpreted as a marker of speech understanding^7,20^. Considering the correlation between feature classes^12^, many studies accounted for the acoustic and lexical segmentation components of linguistic features^7,20^, while others did not^8,42^, potentially measuring the neural tracking of non-linguistic information.

Although the dynamics of the brain responses are known to be non-linear, most of the studies investigating neural tracking relied on linear models, which is a crude simplification. Later research attempted to introduce non-linearity, using deep neural networks. Such architectures relied on simple fully connected layers^14^, recurrent layers^2,32^, or even recently transformer-based architectures^15^. For a global overview of EEG-based deep learning studies see^35^.

Most deep learning work used low-frequency acoustic features, such as the Mel spectrogram, or the speech envelope^2,4^, or higher frequency features such as the fundamental frequency of the voice, f0^34,38^ to improve the decoder’s performance. Although studies using invasive recording techniques showed the encoding of multiple linguistic features^26^, very few EEG-based deep learning studies involved linguistic features^15^. In a previous study^36^, we used a deep learning framework and measured *additional* neural tracking of linguistic features over lexical segmentation features in young healthy native Dutch speakers who listened to Dutch stimuli. This finding emphasized that a component of neural tracking corresponds to the phoneme or word rate, while another corresponds to the semantic context reflected in linguistic features. In addition, linear modeling studies^21,41^ suggested the relationship between understanding and the added value of linguistics.^21^ used two incomprehensible language conditions (i.e. Frisian, a West Germanic language of Friesland, and random-word-shuffling of Dutch speech) to manipulate speech understanding. However, within our deep learning framework, no investigations have been conducted on language data incomprehensible to the test subject.

In this article, we aim to investigate the impact of language understanding on the neural tracking of linguistics using our above-mentioned deep learning framework. Therefore, we fine-tune and evaluate our previously published deep learning framework to measure the added value of linguistics over lexical segmentation features on the neural tracking of three different stimuli: (1) Dutch, (2) Frisian, and (3) scrambled Dutch words. Additionally, we evaluate our model on a language classification task to explore whether our CNN can learn language-specific brain responses.

## Methods

### Participants

In^21^, 19 participants were recruited (6 men and 13 women; mean age ± std = 22 ± 3 years). We included participants that had normal hearing and Dutch as their native language. Participants with attention problems, learning disabilities or sever head trauma were excluded. The latter were identified via a questionnaire. Pure tone audiometry was conducted at octave frequencies from 125 to 8000 Hz to assess the hearing capacity. Participants for whom a hearing threshold exceeded 20 dB HL, were excluded from this study.

### Dataset

The participants listened to a comprehensible story in Dutch, a list of scrambled words in Dutch, and an incomprehensible story in Frisian. The three stories were narrated by the same male native Dutch speaker, who learnt Frisian as a second language. The Dutch story is derived from a podcast series about crime cases, and the Frisian story is a translation of the Dutch. Frisian is a language related to Dutch but poorly understood by Dutch native participants who have no prior knowledge of it. The list of scrambled words consists of randomly shuffled words from the Dutch story. This story plays the role of intermediary comprehension as words are in Dutch (understood), however there is no sentence structure.

The duration of the Dutch, scrambled Dutch and Frisian stories, from now on referred to as “nguage conditions”are 10, 9, and 7 min, respectively. For the Dutch story, the participants listened to it entirely without any break, and had to answer a content question to make sure they paid attention. The Frisian and the scrambled Dutch story were presented in fragments of 2 min, with a word identification task at the end of each fragment to ensure focus. For more details, see^21^.

For the pre-training of our model, we use an additional dataset from^36^, containing EEG of 60 young healthy native Dutch participants listening to 8 to 10 audiobooks of 14 min each.

### Speech features

This study relates 4 features from EEG signals only including the linguistic features that showed a benefit over lexical segmentation features^36^.

The investigated lexical segmentation features are: the onset of any phoneme (PO) and of any word (WO). We then tested the added value of the following linguistic features:

- Cohort entropy (CE), over PO
- Word frequency (WF), over WO

on our model’s performance, measuring the neural tracking of speech.

**Figure 1:**
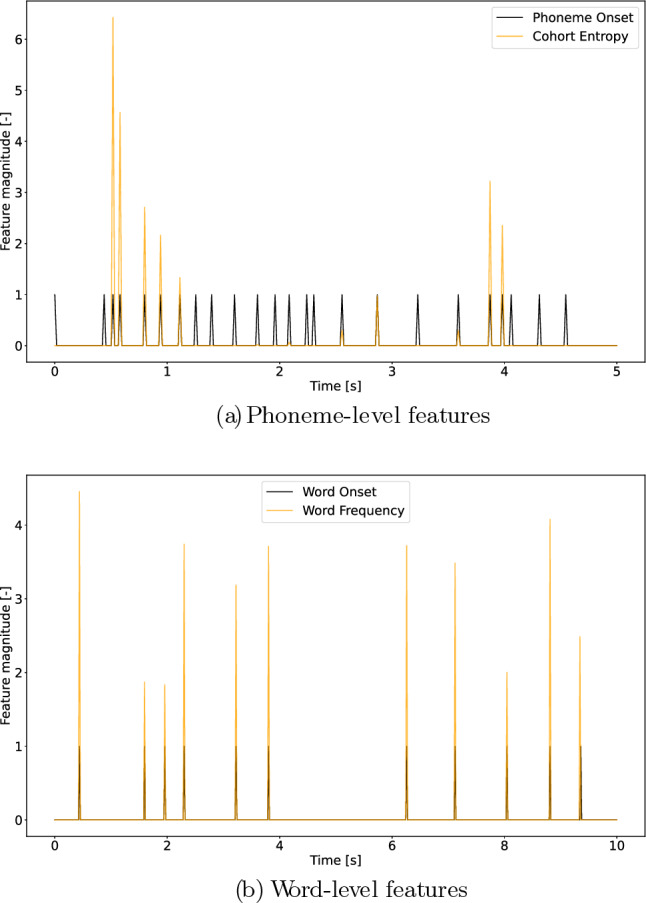
Visualization of word- and phoneme-level lexical segmentation and linguistic features. (**a**) Cohort entropy is depicted in yellow, phoneme onset in black, over a 5 s window. (**b**) Word frequency is depicted in yellow, word onset in black, over a 10 s window.

Example phoneme-level features are depicted in Figure 1a, and word-level features in Figure 1b.

*Lexical segmentation features:* Time-aligned sequences of phonemes and words were extracted by performing a forced alignment of the identified phonemes^19^. PO and WO are the resulting one-dimensional arrays with pulses on the onsets of, respectively, phonemes and words. Silence onsets were set to 0 for both phonemes and words.

*Active cohort of words:* Before introducing cohort entropy, the active cohort of words must be defined. Following previous studies’ definition^7,20^, it is a set of words that starts with the same acoustic input at any point in the word. For example, should we find cohorts in English, the active cohort of words for the phoneme /*n*/ in “ban” corresponds to the ensemble of words that exist in that language starting with “ban” (e.g., “banned”,“bandwidth” etc.). For each phoneme, the active cohort was determined by taking word segments that started with the same phoneme sequence from the lexicon.

*Lexicon:* For the Dutch language, the lexicon for determining the active cohort was based on a custom word-to-phoneme dictionary (9082 words). As some linguistic features are based on the word frequency in Dutch, the prior probability for each word was computed, based on its frequency in the SUBTLEX-NL database^27^.

For the Frisian language, the word-to-phoneme dictionary (75036 words) and the word frequencies were taken from^43^.

*Cohort entropy:* CE reflects the degree of competition among possible words that can be created from the active cohort including the current phoneme. It is defined as the Shannon entropy of the active cohort of words at each phoneme as explained in^7^ (see Equation 1). $CE_{i}$ is the entropy at phoneme *i* and $p_{word}$ is the probability of the given word in the language. The sum iterates over words from the active cohort $cohort_{i}$.

1 $$\begin{aligned} CE_{i}=-\sum _{word}^{cohort_{i}} p_{word}log_{2}(p_{word}) \end{aligned}$$

*Word frequency:* For the Dutch language, the prior probability for each word was based on its frequency in the SUBTLEX-NL database^27^. Values corresponding to words not found in the SUBTLEX-NL were set to 0.

For the Frisian language, the word probabilities were used from^43^. WF is a measure of how frequently a word occurs in the language and is defined in Equation 2.

2 $$\begin{aligned} WF_{i} = -log_{10}\left( p(w_{i})\right) \end{aligned}$$

More details about their implementation can be found in previous studies^6,20,36^.

### Preprocessing

The EEG was initially downsampled using an anti-aliasing filter from 8192 to 128 Hz to decrease the processing time. A multi-channel Wiener filter^37^ was then used to remove eyeblink artifacts, and re-referencing was performed to the average of all electrodes. The resulting signal was band-pass filtered between 0.5 and 25 Hz using a Least Squares filter of order 5000 for the high-pass filter, and 500 for the low-pass filter, with 10% transition bands (transition of frequencies 10% above the lowpass filter and 10% below the highpass) and compensation for the group delay. We then downsampled the signal to 64 Hz.

Lexical segmentation and linguistic features are discrete representation, namely vectors of zero and nonzero values. They were calculated at 64 Hz and no further pre-processing was needed.

### The match-mismatch task

**Figure 2:**
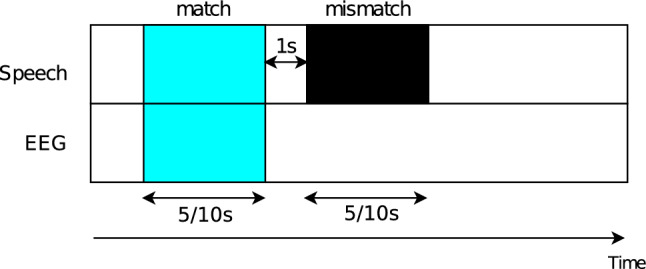
Match-mismatch classification task. The match-mismatch task is a binary classification paradigm that associates the herewith blue EEG and speech segments. The matched speech segment is synchronized with the EEG (blue segment), while the mismatched speech occurs 1 second after the end of the matched segment (black segment). The figure depicts segments of 5 s and 10 s, which will be lengths used in our studies for the phoneme and word levels respectively.

In this study, we use the performance of the match-mismatch (MM) classification task^13^ to measure the neural tracking of different speech features (Figure 2). We use the same paradigm as^36^. The model is trained to associate the EEG segment with the matched speech segment among two presented speech segments. The matched speech segment is synchronized with the EEG, while the mismatched speech segment occurs 1 second after the end of the matched segment. These segments are of fixed length, namely 10 s for word-based features and 5 s for phoneme-based features, to provide enough context to the models as hypothesized by^36^. This task is supervised since the matched and mismatched segments are labeled. The evaluation metric is classification accuracy.

### Multi-input features convolutional neural network^36^

In^36^, we developed a multi-input convolutional neural network (MICNN) model that aims to relate different features of the presented speech to the resulting EEG. The MICNN model has 127k parameters and is trained using binary cross-entropy as its loss function (Adam optimizer, 50 epochs, learning rate: $10^-{3}$). We used early stopping as regularization. It is trained to perform well on the MM task presented in "The match-mismatch task". Through the MM task, the MICNN model learns to measure the neural tracking of speech features, which we can thereafter use to quantify the added value of one speech feature over another. By inputting multiple features, we make sure to account for redundancies and correlations between them and enable interpretation of what information makes the model better on the MM task. In our case, our models enable us to quantify the added value of a given linguistic feature (WF or CE) over their corresponding lexical segmentation feature (WO or PO, respectively)

To ensure that the model has enough data to identify a typical neural response to Dutch linguistic features, we always train the MICNN model on the dataset used in^36^ as a first step. We use an identical training procedure.

### Fine-tuning and evaluation on validation datasets

We performed two fine-tuning conditions: one subject-independent (language fine-tuning) and one subject-dependent (subject fine-tuning). For both, we keep the training parameters mentioned in "Multi-input features convolutional neural network", and solely change the data used for training and evaluation.

For the language condition fine-tuning, we trained a separate model for each subject, including data from the other 25 subjects, for each of the three language conditions (i.e., Dutch, Frisian, and scrambled Dutch): We exclude a selected subject and separate the data from the 25 other subjects in an 60%/20%/20% training/validation/test split. For the 25 other subjects, the first and last 30% of their recording segment were used for training. The first half of the remaining 40% was used for validation (i.e., for regularization) and the second half to get an estimate of the accuracy of unseen speech data. Once the model is fine-tuned, we evaluate it on the selected subject.

For the subject-specific fine-tuning, a 25%/25%/50% training/validation/test split was performed. Compared to the language condition fine-tuning, selecting the data of a single subject divides the amount of data by a factor of 26 (i.e., the number of subjects) for training validation and testing. For the Dutch, Frisian and scrambled Dutch stories, the total amount of data is thus 10 min, 7 min and 9 min respectively. We thus modified the split ratio to increase the amount of data in the validation set to enable keeping the batch size constant across fine-tuning conditions. We used the validation set for regularization. The first and last 12.5% of the recording segment were used for training. The first third of the remaining 75% was used for validation and the two remaining thirds for testing. For each set (training, validation, and testing), each EEG channel was then normalized by subtracting the mean and dividing by the standard deviation.

### Language condition classification

Inspired by the support vector machine (SVM) utilization for aphasia classification from^10^, we use the MM accuracy obtained with four models to classify the language presented to the participant. The four models are the following: the control (word onset or phoneme onset) and linguistic (cohort entropy or word frequency) models for both fine-tuning conditions. We chose to use only the fine-tuned conditions as the non-fine-tuned one was biased towards giving better performance on the Dutch condition. These four MM accuracy values constitute the features provided to the SVM to solve a one-vs-one classification: did the person listen to one or another selected language condition. We consider three language conditions: Dutch, scrambled Dutch, and Frisian, which in total leads to three binary classification tasks.

We used a radial basis function kernel SVM and performed a nested cross-validation approach. In the inner cross-validation, the C-hyperparameter (determining the margin) and pruning were optimized (accuracy-based) and tested in a validation set using 5-fold cross-validation. Predictions were made on the test set in the outer loop using leave-one-subject-out cross-validation. We computed the receiver operating characteristic (ROC) curve and calculated the area under the curve (AUC), and further reported the accuracy, F1-score, sensitivity, and specificity of the classifier.

## Results

### Impact of the language on the neural tracking of linguistic features

We only depict results with language or subject fine-tuning as pure evaluation would potentially give a performance advantage to the model on Dutch because of the pre-training on Dutch stimuli. We still show the non-fine-tuned results in Appendix A.

#### Evaluation of the trained MICNN across languages with language fine-tuning

Although not significant, at both the word and the phoneme levels, the neural tracking when adding the linguistics on top of lexical segmentation features is typically higher at the group level. We depict in Appendix B (see Figure B1a and B1b) the models performances at the phoneme and the word levels across stimuli.

**Figure 3:**
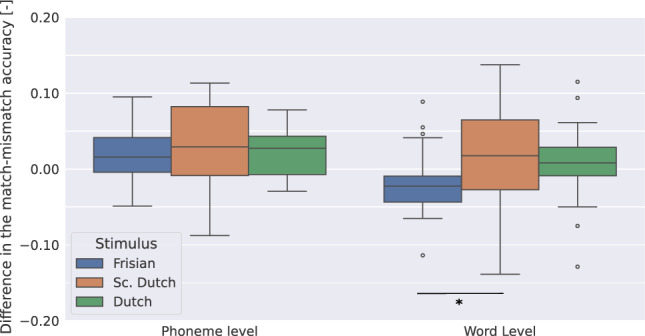
L - C accuracy for three stimuli with a language-finetuned model. L corresponds to the MM accuracy obtained by (1) the cohort entropy model at the phoneme level; (2) the word frequency model at the word level. C corresponds to the MM accuracy obtained by (1) the phoneme onset model at the phoneme level; (2) the word onset model at the word level. *Significance levels*: $p<0.05$: *.

Figure 3 depicts for all three stimuli, the difference in the MM accuracy between L and C conditions for phoneme-level features. We observed no significant difference when comparing the Frisian and the Dutch conditions (Wilcoxon signed-rank test, $W=172, \textit{p}=0.94$), the Dutch and the Sc. Dutch conditions (Wilcoxon signed-rank test, $W=160, \textit{p}=0.73$), and the Frisian and the Sc. Dutch conditions (Wilcoxon signed-rank test, $W=149, \textit{p}=0.51$).

We also depict for all three stimuli, the L-C accuracy at the word level. We observed a significant increase of the L-C accuracy of Sc. Dutch over Frisian (Wilcoxon signed-rank test, $W=97, \textit{p}=0.046$), however no significant difference in the Dutch-Frisian and Dutch-Sc. Dutch comparisons (Wilcoxon signed-rank test, Dutch-Frisian: $W=104, \textit{p}=0.07$, Dutch-Sc. Dutch: $W=163, \textit{p}=0.78$).

To see whether the model could be improved by introducing subject information, we add a subject fine-tuning step in the next Section (for details about the method, see "Fine-tuning and evaluation on validation datasets").

#### Evaluation of the trained MICNN across languages with language and subject fine-tuning

We depict results up to half of the recording length of the shortest stimulus for each subject (i.e., 3.5 min) as we used the other half to fine-tune the model.

**Figure 4:**
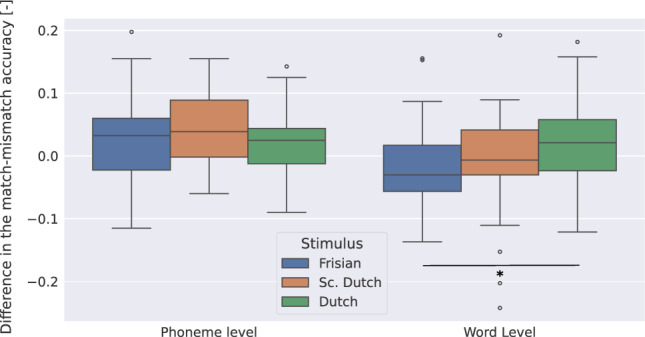
L - C accuracy across recording lengths for three stimuli with a subject-finetuned model. L corresponds to the MM accuracy obtained by (1) the cohort entropy model at the phoneme level; (2) the word frequency model at the word level. C corresponds to the MM accuracy obtained by (1) the phoneme onset model at the phoneme level; (2) the word onset model at the word level. *Significance levels*: $p<0.05$: *.

Figure 4 depicts for all three stimuli, the L-C accuracy at the phoneme level. We observed no significant difference in the L-C accuracy in the Frisian-Sc. Dutch, Dutch-Sc.-Dutch and Frisian-Dutch comparisons (Wilcoxon signed-rank test,$W=141, \textit{p}=0.39$, and $W=118, \textit{p}=0.15$, and $W=152, \textit{p}=0.78$ respectively).

We also depict for all three stimuli, the L-C accuracy at the word level. We observed a significant increase in the L-C accuracy of Dutch over Frisian (Wilcoxon signed-rank test, $W=97, \textit{p}=0.046$). We observed no significant difference in the L-C accuracy for the other comparisons (Wilcoxon signed-rank test, Dutch-Frisian: Dutch-Sc.Dutch: $W=134, \textit{p}=0.30$, and Sc.Dutch-Frisian: $W=144, \textit{p}=0.44$).

### Language classification task

**Figure 5:**
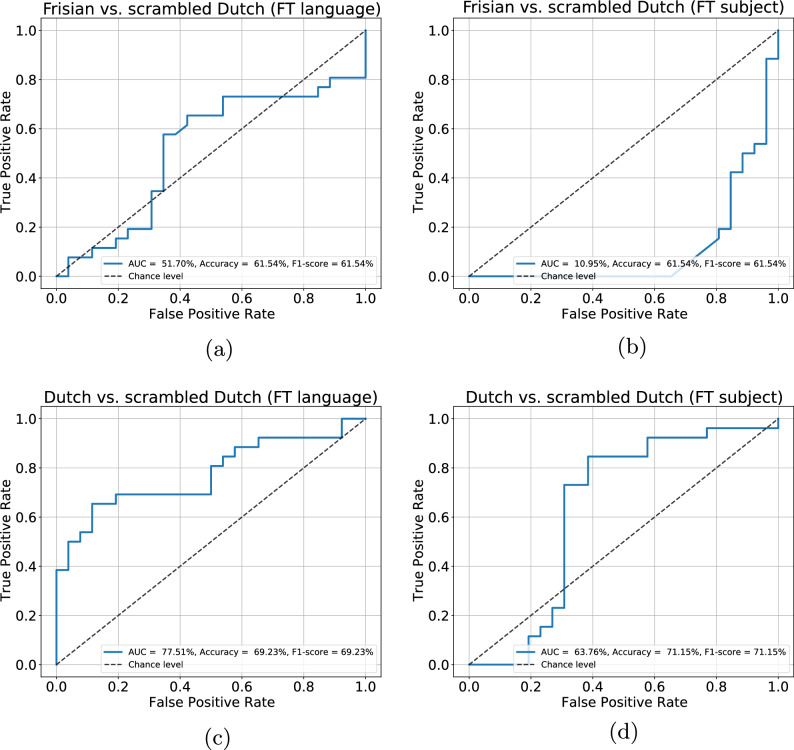
SVM performance across fine-tuning conditions. The performance is depicted for each condition as a ROC curve plotting the true positive rate as a function of the false positive rate. (**a**) Scrambled Dutch vs. Frisian classification with language fine-tuning; (**b**) with subject fine-tuning; (**c**) Scrambled Dutch vs. Dutch classification with language fine-tuning; (**d**) with subject fine-tuning.

Figure 5 depicts the SVM classification results of the three binary classification tasks: (1) Dutch vs. Frisian, (2) Dutch vs. Scrambled Dutch, and (3) Frisian vs. Scrambled Dutch. For more details about the methods, see "Language condition classification".

When evaluated over all subjects, our SVM classifier correctly classified the scrambled Dutch from the Frisian condition with an accuracy of 61.5% for both the FTL and FTS conditions. In addition, the classifier correctly classified the scrambled Dutch from the Dutch condition with an accuracy of 69.23% and 71.15% for the FTL and FTS conditions, respectively. We do not show the results for the Dutch vs. Frisian task, as the classifier performed close to the chance level.

## Discussion

We evaluated a deep learning framework that measures the neural tracking of linguistics on top of lexical segmentation features in different language understanding conditions. Although we used the same dataset, direct comparison with^21^ is difficult, considering the difference in the models, and the features provided to the model.

As our model was trained uniquely on Dutch before, the model might not have learned the typical brain response to Frisian linguistics, or scrambled Dutch, thus leading to overfitting on Dutch, impairing the objective measure of linguistics tracking on other language conditions. To avoid this bias, we fine-tuned our model on Frisian and scrambled Dutch data before respective evaluations.

Since we are interested in the linguistics added value over lexical segmentation features, we compared the difference between the linguistic and lexical segmentation models’ performance across language conditions. For cohort entropy, although there is no significant difference between language conditions in the linguistics added value, the one of Frisian is systematically lower. For word frequency, we observed a significant increase in the added value of linguistics for scrambled Dutch over Frisian. In addition, although not significantly different, the linguistics added value also appeared lower for Frisian than Dutch. This finding suggests that a language that is not understood might show a lower linguistics added value. Regarding the scrambled Dutch results performing non-significantly different than Dutch, although the subjective rating of understanding was very low, individual words are still in Dutch and thus understood. Cohort entropy and word frequency are features that are independent of the order of words in the sentence, which might explain why we do not observe a drop in the neural tracking of linguistics.

Language processing in the brain is influenced by our memory, and top-down processing^23,29^, and might thus have a strong subject-specific component in the response to linguistic features. We therefore decided to fine-tune the models on each subject before evaluation on top of the language fine-tuning. The only significant difference we observed was for word frequency between Dutch and Frisian. This finding supports the conclusion drawn with language fine-tuning: the added value of linguistics is larger when the language is understood. We note that the subject fine-tuning diminished the data used per subject by 50% (i.e., up to  3.5 min of recording) for evaluation, which might not be sufficient to get a good estimate of the accuracy. We therefore do not interpret further the subject fine-tuning condition.

With SVM classifiers, we were able, from the match-mismatch accuracies on our different features, to classify the Frisian vs. the scrambled Dutch condition, as well as the Dutch vs. the scrambled Dutch condition. This suggests that neural tracking of linguistics and lexical segmentation features differs between continuous and scrambled speech. We expected the classifier to be able to differentiate Frisian from Dutch, as participants were not Frisian speakers. Our hypotheses to explain this phenomenon are fourfold: (1) Frisian is a language that is too similar to Dutch to measure a difference in linguistic tracking, therefore there is some understanding by the participants, as emphasized by the subjective rating from^21^ (the authors reported that the speech understanding median subjective rating for the Dutch condition was 100%, while the Frisian and scrambled Dutch were 50% and 10.5%, respectively. The value for Frisian is strangely high and we believe it might in reality be lower.). On the other hand, we believe that within our framework, choosing a similar language is advisable. A very different language (e.g., Mandarin), could have caused a decrease of neural tracking for both lexical segmentation and linguistic features, obliterating our method relying on the added value of linguistics; (2) Linguistic and lexical segmentation features are too correlated, notably because their only differ in the magnitude, which might be too limited to describe the language complexity (3) the magnitude of linguistics has a distribution that tends to be skewed towards the value 1 (i.e., the magnitude of lexical segmentation features) in our three stimuli (see^21^). A more controlled speech content (e.g., sentences with uncommon words) might make the impact of linguistics larger; (4) An additional concern can be added for word frequency: the most frequent words in the language are non-content words (e.g., “and”, “or”, “of”). In addition, most of these words are short words. The model might therefore have learnt a spurious content vs. non-content words threshold from the word frequency, which can be globally narrowed-down to short vs. long words. The length of words can possibly be derived from the word onsets as well. The model could therefore simply use word onset information and omit the magnitude provided by linguistics, which would explain the low benefit of adding word frequency over word onsets.

A possible shortcoming of our training paradigm is the use of a single language for pre-training (i.e., Dutch), which might provide the fine-tuning insufficient abilities to generalize to other language conditions. To solve this issue and preserve a necessary pre-training step for complex deep learning frameworks, we could change our experimental paradigm by: (1) keeping the same language across understanding conditions to avoid biasing the model during pre-training; and (2) avoid random word-shuffling to preserve the word context in sentences. Other non-understanding conditions could involve vocoded speech or degraded-SNR speech as done in^1^. We also evaluated our framework to a speech rate paradigm^41^. However, although we observed a decreased neural tracking of linguistics in challenging listening scenarios (i.e., very high speech rates), we also observed an equivalent decreased neural tracking of lexical segmentation features. We could thus not draw any conclusions whether the nature of this decrease was acoustic or linguistic.

Another pitfall in our comparison across languages is that our linguistic features both rely on word frequencies. The word frequency values were therefore calculated for Dutch and Frisian, respectively. Our participants being Dutch speakers not speaking Frisian, have an language representation in the brain corresponding to the Dutch word frequencies, and not to the Frisian one. This might thus result in a lower neural tracking of linguistics when listening to Frisian content compared to Dutch content.

Linguistic features, as we use them now, are very constrained: they mainly give information about the word or phoneme frequency in the language. Language models are known to capture more information. As an example, the Bidirectional Transformers for Language Understanding (i.e., BERT)^16^ model carries phrase-level information in its early layers, surface (e.g., sentence length) and syntactic (e.g., word order) information in the intermediate layers and semantic features (e.g., subject-verb agreement) in the late layers^25^. Such representations could contain more detailed information about the language than our current linguistic features.^15^ used larger pre-trained speech encoder models, and following up on this work, we could use language model layers, providing information about the structure of language that can be related to brain responses^9,22^.

## Conclusion

In this article, we investigated the impact of language understanding on the neural tracking of linguistics. We demonstrated that our previously developed deep learning framework can classify coherent from nonsense languages using the neural tracking of linguistics. We explored the ability and the limitations of state-of-the-art linguistic features to objectively measure speech understanding using lexical segmentation features as our acoustic tracking baseline. Our findings along with the current literature support the idea that, considering this framework, further work should be dedicated to (1) designing new linguistic features using recent powerful language models, and (2) using incomprehensible and comprehensible speech stimuli from the same language, to facilitate the comparison across conditions.

### Supplementary Information

Below is the link to the electronic supplementary material.

Supplementary Information 1.

## Data Availability

The data that support the findings of this study can be made available from the corresponding author on reasonable request, so far as this is in agreement with privacy and ethical regulations. A subset of the pretraining dataset (i.e., 60 subjects, 10 stories) was published and is available online^5^.
