# Supplementary material for: Classifying coherent versus nonsense speech perception from EEG using linguistic speech features
Source: Sci Rep. 2024 Aug 14;14:18922. doi: 10.1038/s41598-024-69568-0 (PMC11324895; doi:10.1038/s41598-024-69568-0)
Supplement: Supplementary file 1 — Supplementary Information 1. [file 41598_2024_69568_MOESM1_ESM.pdf]

Classifying coherent versus nonsense speech perception from EEG using linguistic speech features

Corentin Puffay[1,2,*], Jonas Vanthornhout[1], Marlies Gillis[1], Pieter De Clercq[1], Bernd Accou[1,2], Hugo Van hamme[2], Tom Francart[1,*]

[1]KU Leuven, Dept. Neurosciences, ExpORL, Leuven, Belgium
[2]KU Leuven, Dept. of Electrical engineering (ESAT), PSI, Leuven, Belgium
[*]Authors to whom any correspondence should be addressed.

E-mail: `corentin.puffay@kuleuven.be`, `jonas.vanthornhout@kuleuven.be`, `marlies.gillis@kuleuven.be`, `pieter.declercq@kuleuven.be`, `bernd.accou@kuleuven`, `hugo.vanhamme@kuleuven.be`, `tom.francart@kuleuven.be`

Appendix A. Evaluation of the trained MICNN across languages without fine-tuning

Figure A1a depicts the MM accuracy obtained for each subject using control (C) and linguistic (L) models across language conditions at the phoneme level. At the group level, no significant differences were found between C and L conditions for the Dutch, scrambled Dutch and Frisian languages. We also depict the difference between L and C models' accuracy across language conditions in Figure A1b. The difference in the match-mismatch accuracy between the linguistic and control model (L-C) was significantly higher for the Sc. Dutch than the Frisian stimuli (Wilcoxon signed-rank test: $W = 97, p = 0.046$).

Figure A2a depicts the MM accuracy obtained for each subject using control (C) and linguistic (L) models across language conditions at the word level. At the group level, no significant differences were found between C and L conditions for the Dutch, scrambled Dutch and Frisian stimuli. We also depict the difference between L and C models' accuracy across language conditions in Figure A2b. The difference in the match-mismatch accuracy between the linguistic and control model (L-C) was significantly higher for the Sc. Dutch than the Frisian stimuli (Wilcoxon signed-rank test: $W = 13, p < 0.001$), and significantly higher for the Dutch than the Frisian stimuli (Wilcoxon signed-rank test: $W = 25, p < 0.001$).

Appendix B. Neural tracking of linguistic over control models across languages

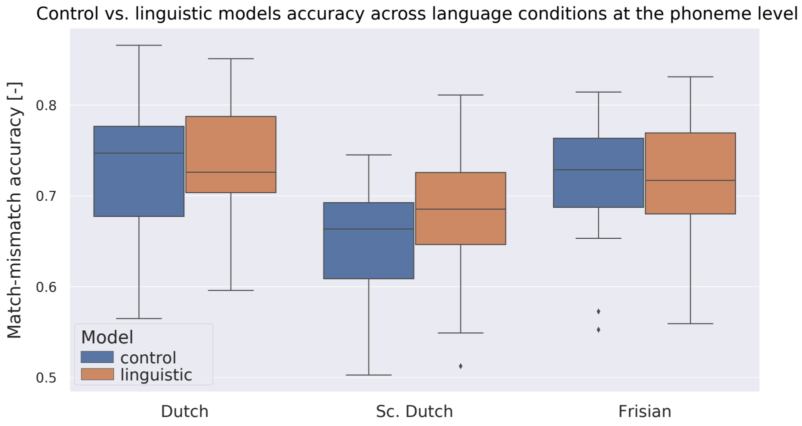

(a) Control vs. linguistic models

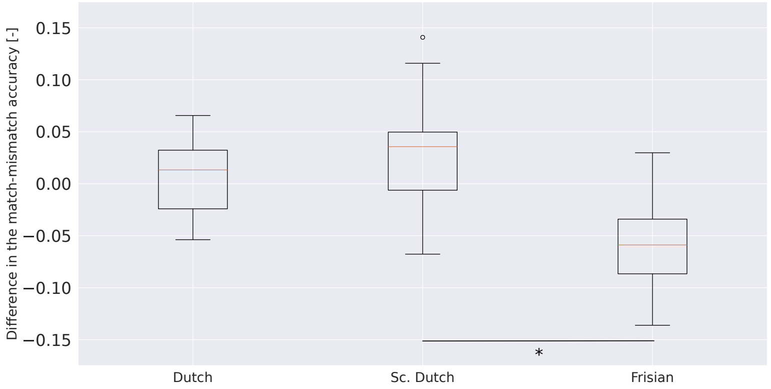

(b) Difference between control and linguistic models

Figure A1: **Control (C) vs. linguistic (L) models' accuracy across language conditions at the phoneme level.** (a) The boxplots represent the control and linguistic model accuracy across language conditions; (b) The boxplot represent for each subject, the difference in accuracy obtained for the linguistic and control models (L-C). The language conditions are Dutch, scrambled Dutch (Sc. Dutch), and Frisian. *Wilcoxon signed-rank test:* $(* : p < 0.05)$

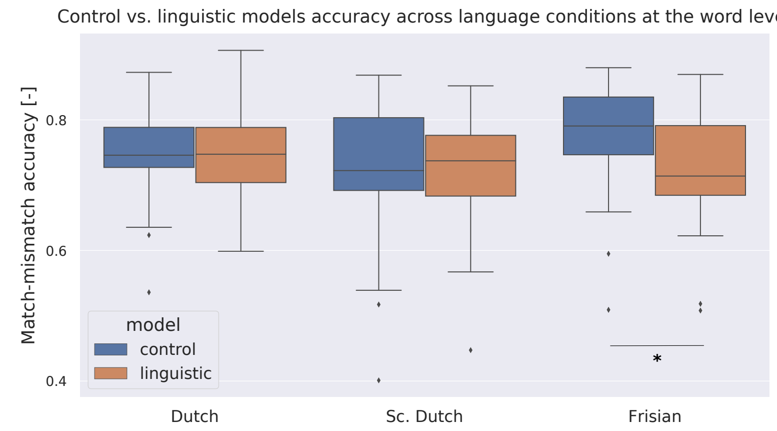

(a) Control vs. linguistic models

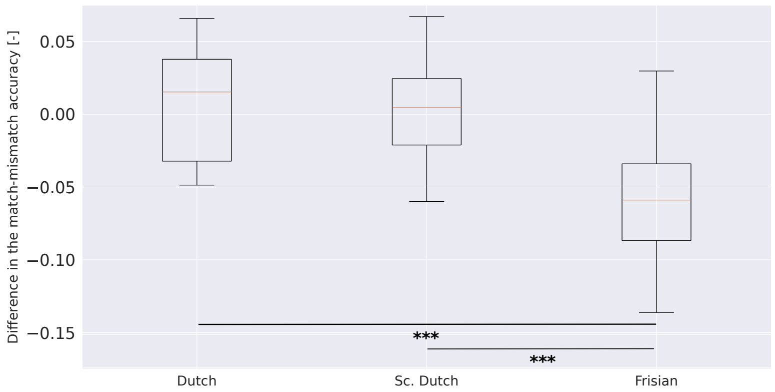

(b) Difference between control and linguistic models

Figure A2: **Control (C) vs. linguistic (L) models' accuracy across language conditions at the word level.** (a) The boxplots represent the control and linguistic model accuracy across language conditions; (b) The boxplot represent for each subject, the difference in accuracy obtained for the linguistic and control models (L-C). The language conditions are Dutch, scrambled Dutch (Sc. Dutch), and Frisian. *Wilcoxon signed-rank test:* $(***: p < 0.001)$

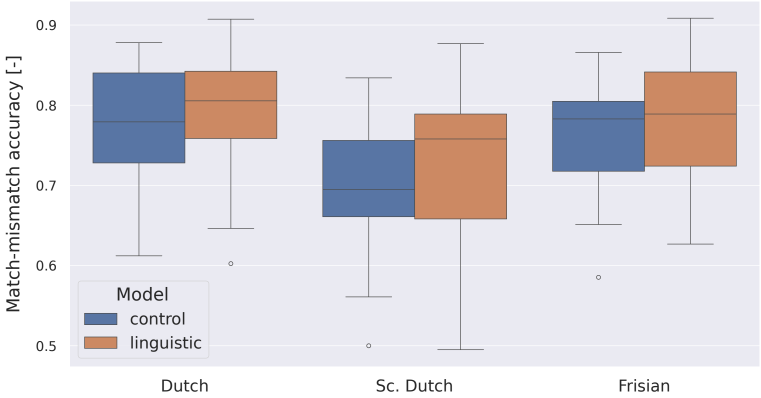

(a) Control vs. linguistic models (phoneme level)

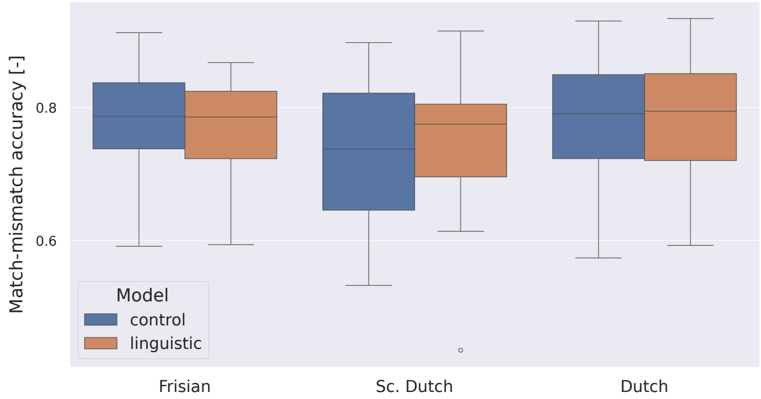

(b) Control vs. linguistic models (word level)

Figure B1: **Control (C) vs. linguistic (L) models' accuracy across language conditions at the phoneme and word level in the language fine-tuning condition.** The boxplots represent the accuracy obtained per subject at (a) the phoneme level (b) the word level. The language conditions are Frisian, scrambled Dutch (Sc. Dutch), and Dutch. *Wilcoxon signed-rank test:* $(* : p < 0.05, ** : p < 0.01, *** : p < 0.001)$
